# The Stability of UV-Defluorination-Driven Crosslinked Carbon Nanotubes: A Raman Study

**DOI:** 10.3390/nano14171464

**Published:** 2024-09-09

**Authors:** Yunxiang Gao, Mohammad Tarequl Islam, Promise Uzoamaka Otuokere, Merlyn Pulikkathara, Yuemin Liu

**Affiliations:** Department of Chemistry and Physics, Prairie View A&M University, Prairie View, TX 77446, USA; mislam8@pvamu.edu (M.T.I.); potuokere@pvamu.edu (P.U.O.); mepulikkathara@pvamu.edu (M.P.); yeliu@pvamu.edu (Y.L.)

**Keywords:** fluorinated carbon nanotubes, defluorination, crosslinking, stability, Raman spectroscopy

## Abstract

Carbon nanotubes (CNTs) are often regarded as semi-rigid, all-carbon polymers. However, unlike conventional polymers that can form 3D networks such as hydrogels or elastomers through crosslinking in solution, CNTs have long been considered non-crosslinkable under mild conditions. This perception changed with our recent discovery of UV-defluorination-driven direct crosslinking of CNTs in solution. In this study, we further investigate the thermal stability of UV-defluorination-driven crosslinked CNTs, revealing that they are metastable and decompose more readily than either pristine or fluorinated CNTs under Raman laser irradiation. Using Raman spectroscopy under controlled laser power, we examined both single-walled and multi-walled fluorinated CNTs. The results demonstrate that UV-defluorinated CNTs exhibit reduced thermal stability compared to their pristine or untreated fluorinated counterparts. This instability is attributed to the strain on the intertube crosslinking bonds resulting from the curved carbon lattice of the linked CNTs. The metallic CNTs in the crosslinked CNT networks decompose and revert to their pristine state more readily than the semiconducting ones. The inherent instability of crosslinked CNTs leads to combustion at temperatures approximately 100 °C lower than those required for non-crosslinked fluorinated CNTs. This property positions crosslinked CNTs as promising candidates for applications where mechanically robust, lightweight materials are needed, along with feasible post-use removal options.

## 1. Introduction

Carbon allotropes are conventionally classified into four categories based on the type of carbon lattice hybridization, including sp^3^-hybridized diamond, sp^2^-hybridized graphitic sheets (such as carbon nanotubes and graphene), fullerenes, and amorphous carbon [1]. The past decades have witnessed significant developments in each type of carbon allotrope and their applications [2,3,4]. Some initial efforts have also been made to design hybrid carbon allotropes by merging existing ones, either through theoretical simulations [5,6,7] or experimental synthesis under extreme conditions [8,9]. For instance, carbon nanotubes (CNTs) have been crosslinked via their carbon lattice under extremely harsh conditions, including high temperatures (~2000 K), high pressures (tens of GPa), or high-energy beam bombardment, to form sp^3^–sp^2^ hybrid carbon nano-allotropes [10,11,12,13,14]. Ion beam irradiation was used to create defects on the surface of carbon nanotubes via *p*-plasma excitation [15], which caused the welding of adjacent tubes via covalent C–C bonds [16]. In contrast to the harsh conditions needed for intertube crosslinking of CNTs, 0D fullerenes (represented by C_60_) with higher lattice curvature can be effectively polymerized in solution under mild conditions with UV irradiation [17]. This distinct difference in the energy needed for self-polymerization is not only attributed to the lattice curvature strain [18] but also to the fact that pristine CNTs are efficient free radical scavengers due to their large delocalized π-electron conjugation systems (“electron sea”) [19].

Despite the “electron sea”, single-walled carbon nanotubes (SWNTs) can be regarded as 1D polymers or ‘the ultimate polymers’ due to their structural definitions [20], physical behaviors in solution [21,22], and rheological properties [23,24]. These similarities in physical properties to conventional polymers, along with evidence of C_60_ photo-polymerization [17], led us to believe that disrupting the delocalized π-electron conjugation systems of CNTs might allow for solution-based crosslinking CNTs under relatively mild conditions, just like conventional polymers.

To disrupt the delocalized π-electron conjugation system of SWNTs, fluorination is a well-known option [25]. Brzhezinskaya et al. also analyzed fluorinated multi-walled carbon nanotubes (MWNTs) and found that fluorination affects both their electronic and atomic structures, resulting in a nearly homogeneous distribution of fluorine throughout the nanotubes with significant changes in both surface and bulk electronic properties [26,27]. These fluorinated carbon nanomaterials have been widely utilized as precursors for further chemical functionalization [28]. In addition, more recent advances highlighted their increasing importance in sustainable technology applications, such as energy storage, catalysis, and fuel cells [29]. This is because the fluorination process introduces two distinct types of C–F bonds: covalent C–F bonds and semi-ionic C–F bonds. The semi-ionic C–F bonds exhibit longer bond lengths and weaker bond energies compared to their covalent counterparts. This feature is advantageous for lithium-ion batteries (LIBs), as it facilitates the reversible breaking and reforming of bonds during cycling [30]. Additionally, the polar nature of C–F bonds in fluorinated carbon materials enhances surface polarity, charge storage capacity, and specific surface area, which improves the performance and cycling stability of supercapacitors [31]. Furthermore, the polarity of C–F bonds has either been leveraged in zinc-ion batteries (ZIBs), where they create zincophilic sites that enhance Zn-ion transfer kinetics [32], or served as a promising alternative to traditional hole transport layer materials for perovskite solar cells (PSCs) [33].

**Figure 1:**
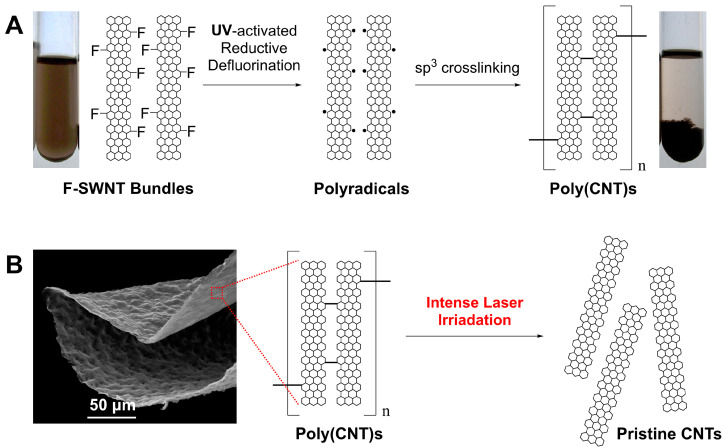
(**A**) Generation and recombination of free radicals in F-SWNT bundles to form poly (CNTs), as described in our previous work [23]. (**B**) Thermal stability of crosslinked CNTs studied in this work.

Another particularly intriguing application of fluorinated carbon lies in carbon lattice engineering. For instance, under extreme conditions of high temperature and pressure, the defluorination of C–F bonds on fluorinated CNTs can lead to the formation of crosslinked carbon nanotubes [34]. Other defluorination efforts have also been applied to remove fluorine from polyvinylidene fluoride (PVDF) using synchrotron radiation, leading to the formation of carbynes [35,36]. These examples demonstrate the significant potential of carbon lattice engineering starting from fluorinated carbon materials. As a representative work in this direction, we successfully crosslinked fluorinated single-walled carbon nanotubes (F-SWNTs) through a UV-defluorination process conducted at room temperature in solution [37]. Using F-SWNTs with a C/F ratio close to 2:1, which led to the complete disruption of the π-conjugated system [38], UV-defluorination allows for the generation of localized reactive free radicals on the carbon backbone. These radicals facilitate crosslinking between nanotubes in solution or within CNT bundles (Figure 1A, or our previous work in reference [37]). However, despite our success in room-temperature crosslinking of CNTs, the thermal stability of these crosslinked CNTs has not yet been investigated. As the role of fluorinated CNTs becomes increasingly more important in renewable energy and sustainable technology sectors, there is an urgent need to understand the stability of directly crosslinked CNTs.

It is noteworthy that crosslinking between the curved CNT surfaces introduces substantial lattice strain, which is known to significantly impact material stability when bond distortion alters the energy landscape of a crystal lattice [39]. This concept has been especially well studied in perovskite solar cells (PSCs), where nanoscale local lattice strains negatively affect the stability of perovskites, accelerating their degradation [40,41,42]. Understanding the relationship between lattice strain and its degradation properties is critical for the development of stable, high-performance perovskite materials [43,44]. Beyond perovskite materials, similar effects have also been observed in carbon lattices. For instance, the C_60_ dimer, C_120_, is significantly less stable and begins to decompose at only 150 °C due to the high curvature strain of the spherical C_60_ [45]. When transitioning from fullerenes to carbon nanotubes, the relatively reduced lattice curvature may enhance thermal stability compared to polymerized fullerenes, though crosslinked CNTs are likely still metastable compared to individual CNTs without strained crosslinking sites. This hypothesis will be explored in this study using Raman spectroscopy under varied laser power.

Raman spectroscopy is a leading tool for characterizing various carbon allotropes through the rotational and vibrational spectral signatures of carbon atoms in their lattice [46]. It is also widely used to assess the thermal stability and degradation of various materials due to its controllable laser power. Examples include Raman laser irradiation studies of red lead [47], synthetic magnetic nanoparticles [48], C_60_ nanowhiskers [49], perovskite nanocrystals [50], and pristine carbon nanotubes [51]. Here, we investigate the stability of UV-defluorination-driven crosslinked single-walled carbon nanotubes (UV-DeF-SWNTs) as well as that of multi-walled carbon nanotubes (UV-DeF-MWNTs) using Raman spectroscopy under controlled laser power. Our findings show that under gradually increased laser power, these UV-defluorinated CNTs undergo significant decomposition, with the crosslinked CNT structure reverting toward that of pristine CNTs (Figure 1B). More interestingly, at increased laser power, a combustion process of both UV-DeF-SWNTs and UV-DeF-MWNTs can be ignited at a lower energy than that required to ignite fluorinated CNTs and pristine CNTs, allowing their potential applications as mechanically robust, lightweight materials with feasible post-use removal options via burning off.

## 2. Materials and Methods

### 2.1. Chemicals and Materials

Fluorinated single-walled carbon nanotubes (F-SWNTs) with an approximate stoichiometry of C_2_F_1_ were purchased from Carbon Nanotechnologies, Inc., Houston, TX, USA. Fluorinated multi-walled carbon nanotubes (F-MWNTs) were obtained from XFNano, Inc., Nanjing, China. Organic solvents, including 99.8% anhydrous *N*, *N*-dimethylformamide (DMF) and 99.8% benzyl alcohol, were purchased from Fisher Scientific. All reagents were used as received.

### 2.2. UV-Defluorination of Fluorinated Carbon Nanotubes

In a typical experiment, F-SWNTs were defluorinated by exposing a DMF solution of F-SWNTs (1 mg/mL) to 254 nm UV irradiation using a 5 W Pen-Ray UV lamp in a photochemical micro-reactor (ACE Glasses) under argon gas purging. After 2 h of irradiation, the product precipitated out of the solution. This precipitate was collected by filtering the solution through a 0.22 μm pore size hydrophilic PVDF membrane filter to form a thin film, which was then rinsed with ethanol and acetone and dried in a vacuum oven for subsequent analysis. Characterization techniques included transmission electron microscopy (TEM), energy dispersive X-ray analysis (EDX), Raman spectroscopy, and thermogravimetric analysis (TGA). UV irradiation of F-MWNTs was conducted under similar conditions, except using benzyl alcohol as the solvent and extending the UV irradiation time to 16 h.

### 2.3. Characterization

TEM images were obtained using a Tecnai G2 F20 S-TWIN microscope (FEI Company, Hillsboro, OR, USA) operating at 200 kV. EDX analyses were conducted with a JEOL JSM-6010LA (JEOL Ltd., Akishima, Tokyo, Japan) at 7 kV utilizing InTouchScope software (version 3.2). Raman spectra at 512 nm were recorded with a Thermo Fisher Scientific DXR3 Raman microscope (Thermo Fisher Scientific, Madison, WI, USA) over a range of 100–2000 cm^−1^, with an adjustable power density of up to 2.9 kW/mm^2^ and a 10× objective lens. Additionally, Raman spectra at 633 nm were collected using a Jobin-Yvon LabRam HR 800 confocal micro-Raman system (HORIBA Scientific, Palaiseau, France), with an adjustable power density of up to 4.2 kW/mm^2^. For both wavelengths, spectra were typically acquired with a 10-s exposure time and 10 accumulations. TGA was performed in the air over a temperature range of 30–800 °C at a scanning rate of 10 °C/min using a TA Instruments TGA 2050 system. Approximately 6 mg of each sample was used, with platinum sample pans utilized for all experiments.

## 3. Results

**Figure 2:**
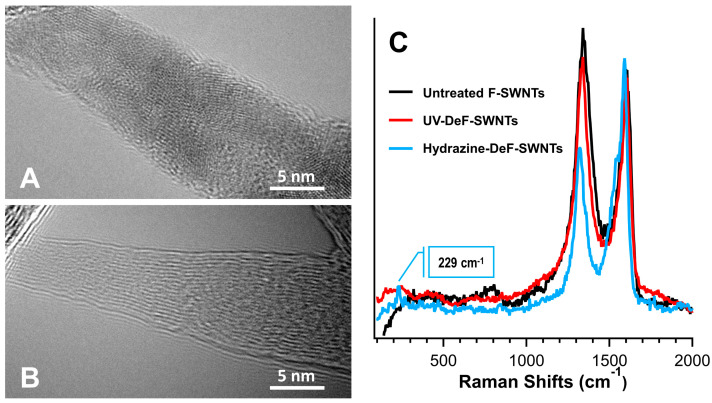
(**A**) TEM images of untreated F-SWNTs and (**B**) UV-crosslinked F-SWNTs. (**C**) Raman spectra of untreated, UV-defluorinated, and hydrazine-defluorinated F-SWNTs, recorded using a 532 nm laser wavelength with a power density of 2.9 kW/mm^2^.

Fluorinated carbon nanotubes dissolve in polar organic solvents due to their dipole interactions [52]. First, directly crosslinked SWNTs were prepared using UV irradiation at 254 nm in DMF solvent with inert argon gas purging, as described in our previous work [37]. This photochemical treatment led to the precipitation of black floccule (Figure 1A), which could not be redispersed again in any solvent, even with the assistance of surfactants, due to defluorination-induced intertube crosslinking. EDX analysis showed a significant decrease in fluorine content in UV-DeF-SWNTs, from 27.27% before to 2.23% after defluorination (Table 1). By contrast, the untreated F-SWNT control sample, which underwent the same solution processing (dissolving, filtrating, drying) but without UV irradiation, retained a higher fluorine content. TEM imaging further illustrates these changes, with the untreated F-SWNT bundles exhibiting a cloudy appearance due to high fluorine surface functionalization (Figure 2A), whereas UV-DeF-SWNTs showed clearer tubular structures due to fluorine removal (Figure 2B).

In general, Raman spectra of SWNTs feature three key bands: the Radial Breathing Mode (RBM), D-band, and G-band. The RBM band, observed in the low-frequency region (100–300 cm^−1^), correlates with the nanotube diameter, offering insights into their size and chirality. The D-band, around 1350 cm^−1^, signals defects and disorder within the carbon lattice, with a higher intensity indicating more diamond-like sp^3^ hybridized carbon atoms. The G-band, near 1580 cm^−1^, corresponds to the tangential stretching mode of the carbon-carbon bonds in the sp^2^ graphene-like carbon lattice of CNTs.

We performed the initial Raman spectroscopy survey for UV-DeF-SWNTs and untreated F-SWNTs using a 532 nm laser at reduced power density (290 W/mm^2^). For comparison, we also defluorinated F-SWNTs using a different method reported by Mickelson et al., which did not crosslink F-SWNTs but instead recovered them toward pristine SWNTs [53]. Because Mickelson’s method involved using anhydrous hydrazine for F-SWNT defluorination, we refer to the resulting product as “HDZ-DeF-SWNTs.” Our Raman results showed that the HDZ-DeF-SWNTs exhibited typical spectral features of partially recovered pristine SWNTs, with a notable decrease in the D-band intensity at 1323 cm^−1^ and the reappearance of the RBM at 229 cm^−1^, which were associated with the effective hydrazine-assisted defluorination (Table 1, fluorine decreasing to 5.11% for HDZ-DeF-SWNTs). By contrast, the UV-DeF-SWNTs yielded a Raman spectrum similar to that of the untreated F-SWNTs, with neither significant D-band decrease nor the reappearance of the RBM, despite having even lower fluorine contents after UV irradiation (2.23% fluorine as shown in Table 1). This result aligned with the observation that UV-defluorination causes intertube crosslinking of neighboring SWNTs in their bundles [37], which maintains the lattice disorder level and the D-band intensity while losing most of the fluorine.

**Figure 3:**
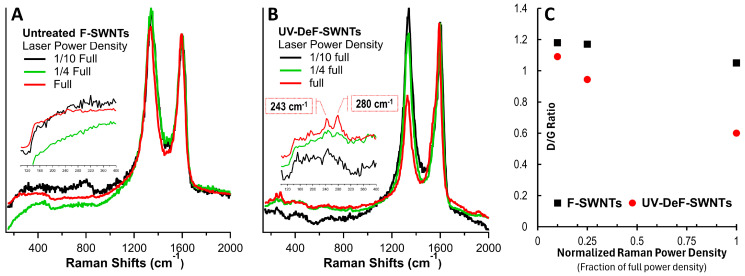
Raman spectra of (**A**) untreated F-SWNTs and (**B**) UV-defluorinated crosslinked F-SWNTs. (**C**) D/G ratio of untreated F-SWNTs and UV-DeF-SWNTs as a function of the fraction of full Raman laser power density. The Raman laser wavelength is 532 nm, with a full laser power density of 2.9 kW/mm^2^. Insets in (**A**,**B**) show amplified RBM bands.

Regarding thermal stability, pristine carbon nanotubes are known for being exceptionally stable due to the strong sp^2^ hybridization of carbon atoms in the carbon lattice, which provides high thermal conductivity and resistance to thermal degradation [54]. However, once polymerized, the formation of sp^3^–sp^3^ intertube carbon bonds between neighboring curved tube surfaces introduces significant lattice strain, leading to potentially decreased thermal stability. To investigate this, we characterized UV-DeF-SWNTs using Raman spectroscopy under varying laser power, with untreated F-SWNTs before UV irradiation as a control. Figure 3 shows these Raman spectra normalized at the G-band peak around 1599 cm^−1^. As can be seen, the untreated F-SWNTs showed no significant responses to the laser power change increasing from 1/10 full to full (Figure 3A). By contrast, the crosslinked UV-DeF-SWNTs displayed a significant decrease in D-band intensity around 1340 cm^−1^ (Figure 3B).

Figure 3C plots the intensity ratio of the D-band to the G-band, *I_D_/I_G_*, versus the varying Raman laser power. For the untreated F-SWNT control samples, a slight decrease in *I_D_/I_G_* was observed with increasing laser power. This trend was consistent with the effects of thermal annealing, which partially removed fluorine from the F-SWNTs, thereby reducing the structural disorder introduced by fluorination [55]. However, the slight decrease indicated that the thermal effect induced by laser radiation was much less significant than that of conventional furnace annealing. By contrast, the UV-defluorinated crosslinked SWNTs (UV-DeF-SWNTs), shown in Figure 3B,C, exhibited a substantial decrease in *I_D_/I_G_* in response to increasing laser power density—from 1.1 at 0.29 kW/mm^2^ to 0.6 at 2.9 kW/mm^2^. This suggested that the crosslinked SWNTs underwent more pronounced thermal decomposition under these conditions, progressively reverting toward de-crosslinked, pristine nanotubes. The greater instability of directly crosslinked SWNTs is likely due to curvature strain, similar to the decomposition of strained crosslinking bonds observed in other polymerized carbon nanostructures, such as Poly(C_60_), under intense Raman laser irradiation [56].

More evidence was observed in the RBM band region (200–300 cm^−1^), which is the characteristic of the radial vibration of the carbon atoms in a perfectly cylindrical nanotube. Fluorination introduces sp^3^ hybridization, distorting the cylindrical carbon lattice and making RBM bands absent in untreated F-SWNTs (Figure 3A, inset). These bands of the control samples did not reappear with increased laser power density. However, in the crosslinked UV-DeF-SWNTs, the RBM bands reappeared at higher laser power densities (Figure 3B, inset), which was consistent with the elimination of fluorine atoms (Table 1) and the decrease in the D-band (Figure 3B), indicating a de-crosslinking process with recovery toward pristine SWNTs. Further evidence was seen in the RBM band region (200–300 cm^−1^), characteristic of the radial vibration of carbon atoms in perfectly cylindrical nanotubes. Fluorination introduced sp³ hybridization, distorting the cylindrical carbon lattice and causing the RBM bands to disappear in untreated F-SWNTs (Figure 3A, inset). These bands remained absent in the control samples, even with increasing laser power density. However, in the crosslinked UV-DeF-SWNTs, the RBM bands reappeared at higher laser power densities (Figure 3B, inset). This reappearance was consistent with the removal of fluorine atoms (Table 1) and the corresponding decrease in the D-band (Figure 3B), suggesting a de-crosslinking process and recovery toward pristine SWNTs.

RBM bands also provide diameter information, as the RBM frequency (ω_RBM_) is inversely proportional to the nanotube diameter (*d_t_*), following the relationship ω_RBM_ = 248/*d_t_* [57]. The inset in Figure 3B shows that at full laser power, UV-DeF-SWNT samples exhibited recovered RBM peaks at 280 and 243 cm^−1^, corresponding to diameters of 0.88 nm and 0.98 nm, respectively. The Kataura plot correlates SWNT diameter with their electronic properties and chiralities. Under 532 nm laser excitation (2.33 eV), RBM peaks between 275 and 200 cm^−1^ (0.90~1.24 nm in diameter) are from metallic nanotubes, while those above 275 cm^−1^ (below 0.90 nm in diameter) are from semiconducting tubes [58]. Thus, the 243 cm^−1^ peak in Figure 3B indicated metallic tubes, and the 280 cm^−1^ peak represented semiconducting tubes. Interestingly, under 1/4 full laser power, the metallic RBM peak at 243 cm^−1^ was stronger than the semiconducting peak at 280 cm^−1^; however, the semiconducting peak increased significantly at full power, suppressing the metallic peak. This suggested that metallic SWNTs decomposed and recovered more readily than semiconducting ones in the crosslinked SWNT bundles or networks, consistent with observations in pristine SWNTs, where metallic tubes are more prone to Raman laser damage than semiconducting tubes [59]. This behavior is likely due to the higher density of free electrons in metallic SWNTs, which enhances electron–phonon coupling and photon energy absorption that leads to more localized heating and increased structural instability.

**Figure 4:**
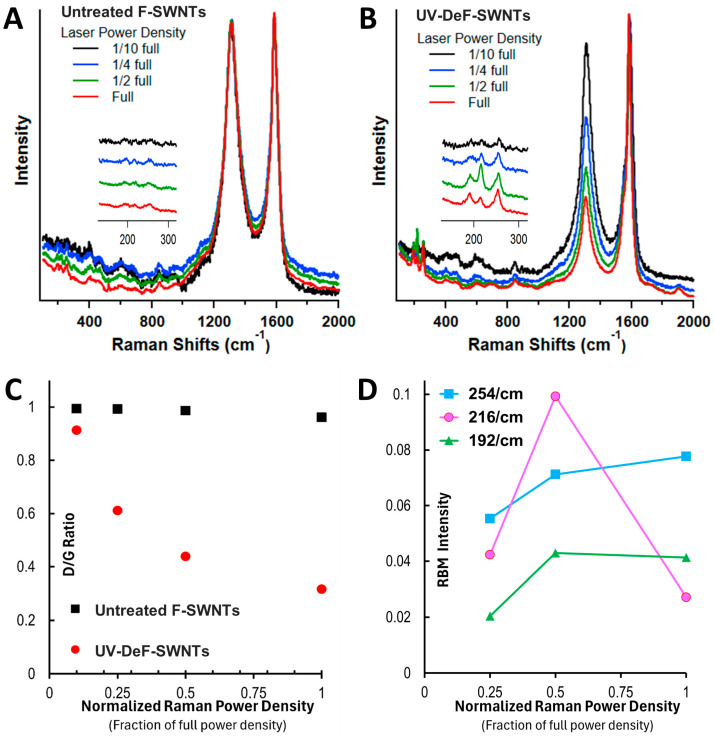
Raman spectra of (**A**) untreated F-SWNTs and (**B**) crosslinked F-SWNTs via defluorination, using a 633 nm laser at varying power densities. (**C**) D/G ratio of untreated and crosslinked F-SWNTs as a function of the fraction of full laser power density. (**D**) Changes in RBM peak intensity in response to increasing laser power density. Full power density: 4.2 kW/mm^2^.

The RBM frequency of each individual SWNT is not directly altered by the laser excitation wavelength, but the excitation wavelength does determine which chiralities of SWNTs are activated, selectively enhancing certain RBM frequencies in the Raman spectrum. Thus, we further analyzed the UV-DeF-SWNT and untreated F-SWNT control samples using 633 nm laser excitation at varied laser power densities. Figure 4 shows that untreated F-SWNTs exhibited no significant changes across different laser power densities (Figure 4A). By contrast, the crosslinked UV-DeF-SWNTs showed a significant decrease in D-band intensity (Figure 4B) and the *I_D_/I_G_* ratio (Figure 4C) with increasing laser power density, indicating thermal decomposition of the crosslinked SWNTs and their recovery toward de-crosslinked pristine nanotubes.

RBM bands did not reappear in untreated F-SWNTs when using 633 nm laser excitation across all applied laser power densities (Figure 4A, inset). However, in UV-DeF-SWNTs, RBM bands emerged at 254, 216, and 192 cm^−1^ at increased power, corresponding to 0.98, 1.15, and 1.29 nm diameter tubes, respectively (Figure 4B, inset). According to the Kataura plot, at 633 nm excitation (1.96 eV), RBM bands below 225 cm^−1^ indicate metallic nanotubes, while those above 225 cm^−1^ are from semiconducting nanotubes [60]. Applying this to our 633 nm Raman results, the RBM peaks at 216 and 192 cm^−1^ corresponded to metallic SWNTs, and the peak at 254 cm^−1^ to semiconducting SWNTs. The peak intensity of the semiconducting SWNT at 254 cm^−1^ continuously increased with rising laser power (Figure 4D), consistent with the behavior observed with a 532 nm laser (Figure 3B), suggesting that the recovered semiconducting SWNTs from the laser thermal decomposition survived under increased laser power. By contrast, the RBM peak intensity of metallic SWNTs at 216 and 192 cm^−1^ initially increased with power density increasing from 1/10 full to 1/2 full, but started to decrease when the laser power further reached full power. This indicated that metallic tubes were more easily decomposed from the crosslinked state, reverting to individual pristine SWNTs, and that they were more susceptible to damage from Raman laser irradiation than semiconducting tubes [59]. Notably, the peak intensity of the smaller diameter metallic tubes at 216 cm^−1^ decreased faster than that of the larger metallic tubes at 192 cm^−1^, agreeing with previous findings that smaller diameter pristine metallic nanotubes are more prone to thermal degradation [61].

**Figure 5:**
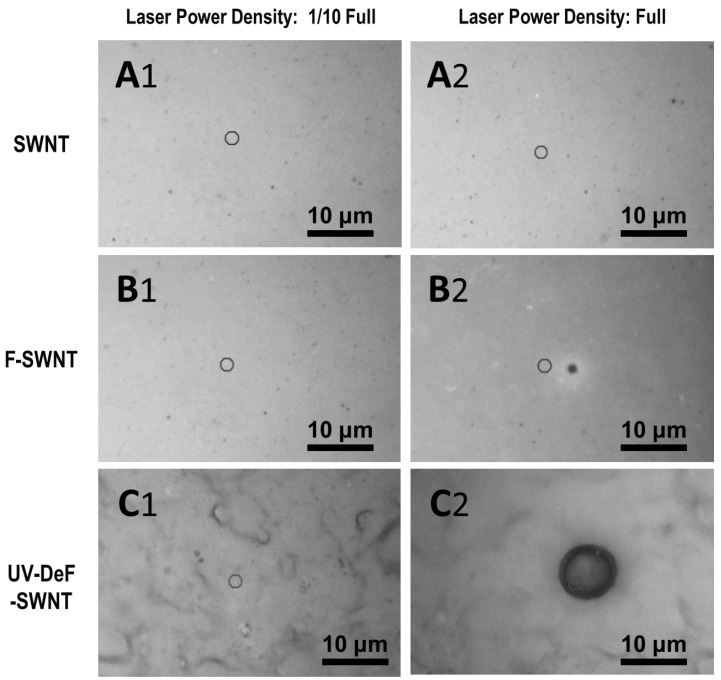
Microscope images of the Raman laser-irradiated sample areas: (**A1**,**A2**) pristine SWNTs, (**B1**,**B2**) untreated F-SWNTs, and (**C1**,**C2**) crosslinked UV-DeF-SWNTs irradiated at 1/10 (Column 1) and full (Column 2) power density, respectively. Full power density: 2.9 kW/mm^2^.

During the Raman spectroscopy characterization, we captured microscope images of the sample areas irradiated by the Raman laser spot. Figure 5(A1,A2) presents the images of pristine SWNTs irradiated with a 532 nm Raman laser at 1/10 full power and at full power, respectively. There was no visible damage to the pristine SWNTs, even at full laser power. By contrast, for untreated F-SWNTs, no damage was observed at 1/10 full laser power (Figure 5(B1)); however, a hole was drilled at full power due to stronger thermal effects (Figure 5(B2)). The size of the burned area was approximately 2 μm, nearly matching the laser spot size. The comparison between Figure 5(A1,A2,B1,B2) indicated that pristine SWNTs have higher thermal stability than fluorinated ones. Interestingly, although the crosslinked UV-DeF-SWNTs also showed no burning spot at 1/10 full power density (Figure 5(C1)), the burned area was significantly larger than the laser spot size at full power density (Figure 5(C2)). This suggested that the laser may have ignited the crosslinked SWNTs, causing the burned area to expand rapidly, which was likely due to the Raman characterization being performed in the presence of air.

Following the study on fluorinated single-walled carbon nanotubes, we extended the work to fluorinated multi-walled carbon nanotubes (F-MWNTs) using the same UV irradiation method and Raman characterization techniques. We found that UV irradiation of F-MWNTs only resulted in partial defluorination. We note the product of these UV-defluorinated F-MWNTs as UV-DeF-MWNTs. As shown in Table 2, the fluorine content decreased from 44.18% by atom counts in the untreated F-MWNT samples to 28.42% in UV-DeF-MWNTs after 16 h of UV exposure, representing a loss of approximately 48% of the original fluorine content after calibrating the total atom number in UV-DeF-MWNTs by considering the loss of fluorine. This indicated that F-MWNT defluorination was significantly less efficient than the near-complete defluorination observed in F-SWNTs, where the fluorine content decreased to 2.23% within just 2 h of UV irradiation. The lower and slower defluorination rate in F-MWNTs is likely due to the less pronounced curvature of the MWNT carbon lattice compared to the highly curved SWNTs [62]. Additionally, similar to F-SWNTs, F-MWNTs showed a slight increase in oxygen content post-UV irradiation, which could be attributed to the presence of O_2_ contaminants in an imperfect solvation system [63] or trace amounts of water from polar organic solvents [64].

We characterized the untreated F-MWNTs and UV-DeF-MWNTs via Raman spectroscopy only using one wavelength, the 532 nm Raman laser, since the purpose of varying the Raman laser wavelength was mainly for observing the RBM vibration in SWNTs of different diameter and chirality. For MWNTs, the RBM bands are naturally absent because the motion of the outer tubes is highly constrained by the multilayer interactions with the inner graphitic layers. Thus, we also only used the intensity ratio of the D-band to the G-band, *I_D_/I_G_*, as a measure of the defect degree in MWNT-related samples.

**Figure 6:**
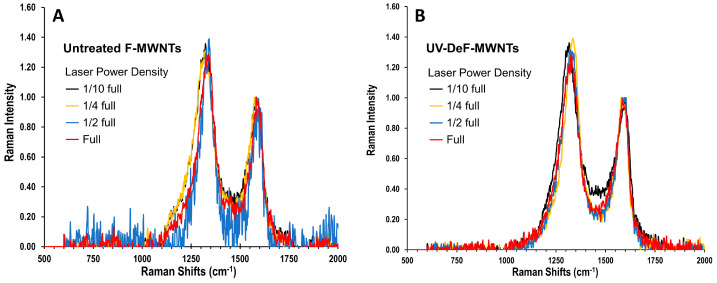
Raman spectra of (**A**) untreated F-MWNTs and (**B**) UV-DeF-MWNTs. Laser wavelength: 532 nm, with a full power density of 2.9 kW/mm^2^.

As shown in Figure 6A,B, with the G-band normalized to 1 and the laser power density reduced to 1/10 full power to avoid thermal damage, both untreated and UV-fluorinated F-MWNTs exhibited a similar *I_D_/I_G_* ratio of 1.4. For MWNTs, the *I_D_/I_G_* ratio typically increases after surface chemical functionalization and decreases when MWNTs recover toward their pristine state [65]. Thus, compared to the untreated F-MWNTs, the unchanged *I_D_/I_G_* ratio of UV-DeF-MWNTs after 48% fluorine elimination indicated that the defluorination process did not primarily restore sp^2^ bonding within the carbon lattice but rather aligned with the formation of intertube-crosslinking sp^3^ carbon bonds. This result conflicted with previous studies on FCNT defluorination via conventional thermal annealing methods [25,27], where defluorination leads to the partial recovery of pristine CNTs. Such a difference is likely due to the defluorination method used. In polar organic solvents, fluorine atoms on the CNT surface may form hydrogen bonds with other solvated species in the medium (such as trace amounts of dissolved H_2_O). Our UV excitation-based defluorination method specifically excites and breaks the C–F bonds, even when the released fluorine atoms have interactions with hydrogen donors via hydrogen bonds, which stabilizes the UV-eliminated fluoride and reduces the needs of the neighboring fluorine atom to be eliminated simultaneously. This fluorine elimination process leaves individual carbon radicals on the surface of the CNTs (as shown in Figure 1A or our previous work [37]), facilitating intertube crosslinking. By contrast, during thermal annealing conducted under dry conditions, the fluorine atoms on the CNT surface do not form pre-complexed hydrogen bonds with solvated hydrogen donors. Instead, their elimination needs simultaneous binding with neighboring fluorine atoms, leading to the recovery of pristine carbon nanotubes.

**Figure 7:**
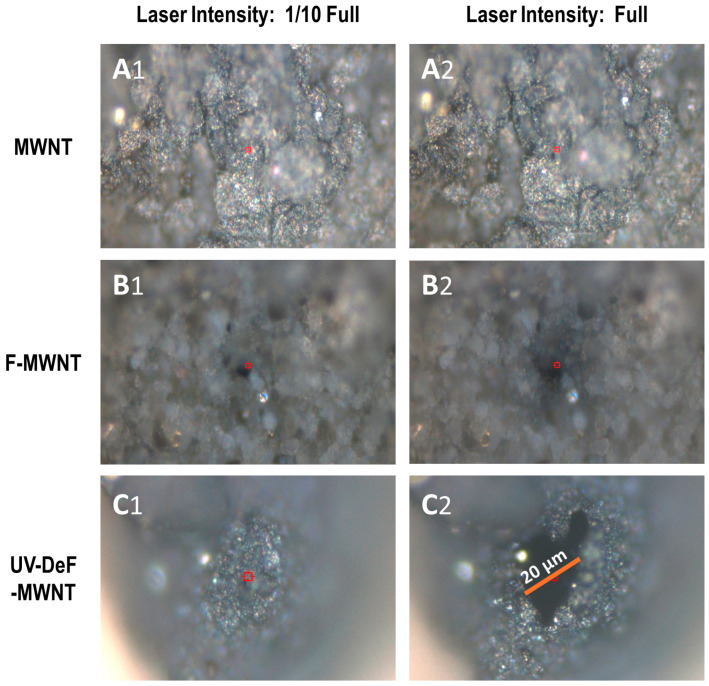
Microscope images of various MWNTs irradiated with a 532 nm Raman laser at 1/10 full power density (Left, Column 1, **A1**,**B1**,**C1**) and full power density (Right, Column 2, **A2**,**B2**,**C2**). (**A1**,**A2**) Pristine MWNTs, (**B1**,**B2**) Untreated F-MWNTs, and (**C1**,**C2**) UV-defluorinated MWNTs (UV-DeF-MWNTs).

The untreated F-MWNTs did not show a decrease in the D-band intensity across the laser power range from 1/10 full to full (Figure 6A), similar to the behavior observed in untreated F-SWNTs (Figure 3A). Notably, UV-DeF-MWNTs also did not show a decrease in the D-band intensity within the same power density range (Figure 6B), which differed from the trends seen in UV-DeF-SWNTs, indicating higher thermal stability of UV-DeF-MWNTs compared to UV-DeF-SWNTs due to the reduced curvature strain on MWNTs. Figure 7 shows the microscope images of various MWNT-based samples irradiated with a 532 nm Raman laser at 1/10 full power density (Figure 7, left column) and full power density (Figure 7, right column), respectively. MWNTs, due to their multi-layer coherent structure, are more rigid than SWNTs. This rigidity caused MWNT films prepared by the filtration procedure to show less resistance to shrinkage during the solvent drying process, resulting in a more uneven film surface under a microscope.

Figure 7(A1,A2) shows the microscopic images of pristine MWNTs irradiated with a 532 nm Raman laser at 1/10 full power and full power, respectively. There was no visible damage to the pristine MWNTs, even at full laser power (Figure 7(A2)). For untreated F-MWNTs, both spots exposed to 1/10 power density (Figure 7(B1)) and full power density (Figure 7(B2)) exhibited small burning holes equivalent to the laser spot size, indicating that fluorinated MWNTs were thermally less stable than their pristine counterparts. Comparatively, F-MWNTs showed a laser-drilled hole under 1/10 of full power (Figure 7(B1)), whereas F-SWNTs exhibited almost no damage under similar conditions (Figure 5(B1)), suggesting that the commercially available F-MWNTs used in this study were slightly less thermally stable than F-SWNTs. This is likely due to the higher fluorination level in the commercially available untreated F-MWNTs (44.18% F) compared to F-SWNTs (27.79% F), resulting in a higher defect level that reduces the thermal stability of F-MWNTs more significantly.

On the other hand, for UV-DeF-MWNTs, although laser radiation at 1/10 full power did not cause noticeable damage to the samples (Figure 7(C1)), at full laser power, the laser-burned area was significantly larger than the laser spot size (Figure 7(C2)), suggesting that the laser may have ignited combustions in the UV-DeF-MWNT sample, causing the burning area to expand rapidly. To confirm the occurrence of combustion, we performed TGA in air, anticipating that the TGA curve may show a typical featured change when combustion is ignited. For example, a rapid rise in the sample temperature may potentially outpace the instrument’s programmed heating rate, resulting in a temperature spike, and after the exothermic event (when the combustion process stops), the temperature returns to the temperature-increasing baseline set by the heating program.

**Figure 8:**
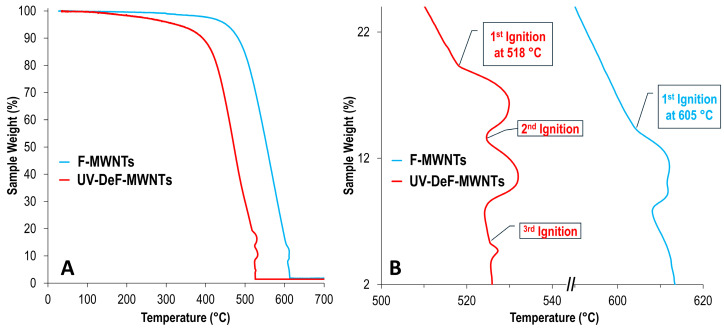
(**A**) TGA curves of F-MWNTs and UV-DeF-MWNTs. (**B**) Enlarged view of the ignition and combustion region featured in (**A**).

As expected, small temperature bumps were observed in the TGA curve of UV-DeF-MWNTs slightly above 500 °C, indicating rapid exothermic processes, while for untreated F-SWNTs, such bumps were not observed until the temperature went beyond 600 °C (Figure 8). These TGA features aligned with the observation that full-power UV irradiation of untreated F-SWNTs did not result in a larger damaged area (Figure 7(B2)), whereas the same irradiation of UV-DeF-MWNTs produced a significantly larger burnt area than the actual laser spot size (Figure 7(C2)). Notably, these TGA bumps suggested exothermic processes causing a temperature spike without a corresponding sharp mass loss. This can be attributed to the solid packing of crosslinked nanotubes. Combustion on the crosslinked CNT film surface rapidly raised the temperature of the sample to cause sharp temperature spikes in the TGA curve, but the mass loss was a slower process due to the tight crosslinking-caused packing. After these exothermic events, the TGA curves returned to the programmed, heating rate-controlled temperature baseline, forming the bumps featured in the TGA curve (Figure 8B). Based on the first combustion-related TGA bump that appeared during the heating process, the UV-DeF-MWNTs could be ignited at significantly lower temperatures, by about 100 °C, compared to untreated F-SWNTs.

## 4. Conclusions

In summary, we successfully characterized the thermal stability of crosslinked carbon nanotubes (CNTs) prepared via a UV-defluorination method, as introduced in our previous work [37]. Both single-walled (UV-DeF-SWNTs) and multi-walled (UV-DeF-MWNTs) nanotubes were examined. Controlled Raman spectroscopy experiments revealed that the crosslinked UV-DeF-SWNTs and UV-DeF-MWNTs remained stable at low laser power densities but began to decompose at higher power densities. Compared to non-crosslinked pristine or fluorinated CNTs, the crosslinked CNTs exhibited more pronounced thermal decomposition under the same laser irradiation, progressively reverting toward de-crosslinked, pristine nanotubes. This increased instability of crosslinked CNTs is attributed to the lattice strain imposed on the intertube sp^3^ crosslinking bonds by the curved CNT surface. Notably, metallic CNTs within the crosslinked networks de-crosslinked and recovered more readily than semiconducting CNTs, but they could also be burned out first if the laser power kept increasing. Under full laser power, the UV-defluorinated nanotubes showed signs of combustion, a phenomenon not observed in pristine or untreated fluorinated CNTs under the same conditions. Thermogravimetric analysis further confirmed laser-triggered combustion in the crosslinked CNT samples, with UV-DeF-MWNTs showing small combustion peaks around 518 °C—nearly 100 °C lower than those of untreated F-MWNTs. This suggests a lower energy barrier for decomposition of the crosslinked CNTs, likely due to the metastable nature of the crosslinked sites. The significantly larger burn areas observed under full-power Raman laser irradiation, compared to pristine or untreated fluorinated CNTs, reinforced this conclusion.

Finally, the ability to create mechanically robust, lightweight carbon materials through CNT crosslinking and subsequently degrade them under specific conditions (such as laser irradiation or thermal treatment) opens new avenues for applications requiring high-performance materials with sustainable disposal options. Future research should explore potential applications of UV-defluorination-crosslinked CNTs in structural reinforcement for advanced composites and energy-related technologies, where the material’s strength-to-weight ratio and controlled degradation are crucial.

## Figures and Tables

**Table 1:** EDX composition of F-SWNT samples treated under various conditions.

Samples	Conditions	Composition (atom%)			
		C	O	F	N
Untreated F-SWNTs	No UV	68.23	3.98	27.79	0.00
UV-DeF-SWNTs	UV, 2 h	92.66	5.11	2.23	0.00
HDZ-DeF-SWNTs	Hydrazine, 2 h	85.37	4.43	5.71	4.50

**Table 2:** SEM-EDX composition of F-MWNT samples treated under various conditions.

Samples	Conditions	Composition (atom%)		
		C	O	F
F-MWNTs	Untreated	54.15	1.67	44.18
UV-DeF-MWNTs	UV, 16 h	66.59	4.99	28.42

## Data Availability

The data are available from the corresponding author upon reasonable request.
